# Need to Perform Rehabilitation Exercises at Home by Parents of Children with Neurological Diseases to Maintain Performance During COVID-19 Lockdown

**DOI:** 10.22037/ijcn.v16i1.30019

**Published:** 2021

**Authors:** Alireza SHAMSODDINI, Behzad BAZIGAR, Hamid DALVAND

**Affiliations:** 1 Exercise PhysiologyResearch Center, LifestyleInstitute, Baqiyatallah University of Medica Sciences, Tehran, Iran.; 2 Department of occupationatherapy, Faculty ofrehabilitation, Tehran University of MedicalSciences, Tehran, Iran

**Keywords:** Home-Based exercises, Rehabilitation, Cerebral palsy, Neurologic diseases, COVID-19

## Abstract

With the outbreak of the novel pandemic coronavirus disease 2019 worldwide, numerous pediatric rehabilitation clinics have closed to support social and physical distancing, and therapists similar to other individuals are staying at their homes. There is a common concern of parents and caregivers that how and with what quality children’s rehabilitation exercises should proceed. Most children with neurological diseases have problems, such as muscle spasticity, range of motion (ROM) limitation, muscle shortening, balance loss, and mobility and movement impairments. The normalization of muscle tone, preservation of ROM, muscle stretch, and improvement of fine and gross motor skills and balance are essential activities that need to be considered. Therefore, this study aimed to summarize the necessities of a home-based rehabilitation exercise program during the quarantine period.

## Introduction

Since the end of 2019, due to the outbreak of coronavirus disease 2019 (COVID-19) in China and its subsequent epidemic around the world, which is a deadly disease, a public health emergency has been created (1). According to the World Health Organization, this lethal virus is transmitted among individuals through direct, indirect (i.e., contaminated objects or surfaces), or close contact with infected individuals via mouth and nose secretions, especially if individuals have close contact with each other or if individuals cough or sneeze .(1, 2) 

Preventive actions, such as quarantine and no contact or minimization of contact with others, may effectively limit the rapid spread of this virus (2). Although COVID-19 affects the adult population (87%), recent findings have demonstrated 2% of its spread among children of 19 years and younger (3). A preliminary study conducted by Ling Mao et al. in Wuhan, China, reported that about one-third of patients showed some degree of involvement in neurological disorders (4). 

Neurological disease studies have shown that brain dysfunction is often associated with immunological dysregulation. Therefore, it is probable that children with neurological disorders are even at higher risk of COVID-19 spreading than other individuals in the community (5). Due to the social distancing, lockdown, or quarantine strategy selected in most countries in combating the COVID-19 pandemic, as other occupations and social service activities, rehabilitation centers have been closed; consequently, parents have not had access to rehabilitation services. Therefore, parents have to do the exercises and rehabilitation activities themselves at home or receive them online from therapists and perform them for their children. 

Children with long-term conditions need exercise-based rehabilitation to improve fitness and function (6). Prescribed exercise programs often comprise a part of home-based rehabilitation or self-management for long-term conditions and are typically unsupervised by therapists (7). In home-based rehabilitation exercises, family members are trained on individualized exercises that help children with neurological disorders (8). However, there are some questions, such as under these situations, which types of rehabilitation exercises and activities would be helpful or contraindicated for these children by parents? Which aspects of children’s rehabilitation exercises and activities are essential priorities?

The most important goals of home-based rehabilitation exercises and activities programs for children with neurologic diseases (9-13) are as follow:

1. Decreasing muscle tonicity

2. Maintaining joints range of motion (ROM) 

3. Preventing shortening of muscles with stretching exercises

4. Maintaining gross and fine motor activities

5. Maintaining and improving balance abilities

This study was based on searching the literature in PubMed, ISI Web of Science, Scopus, and Google Scholar using a key phrase, namely “Home-Based Rehabilitation Program for Children with Neurologic Diseases”, with the emphasis on clinical studies. The current assessments also rest on the researchers’ own clinical experience and research at Baqiyatallah Hospital in Tehran, Iran. 

**Literature Review**

There are epidemiological, clinical, and review studies about home-based rehabilitation programs for children with neurological diseases. This study will briefly and concisely discuss how to perform these home-based rehabilitation exercises and activities.

**Decreasing Muscle Tonicity**

More than 80% of children with cerebral palsy develop spasticity, which is a constant contraction of muscles. When muscles contract, they constantly overstretch the joints, which limits ROM. As children are constantly growing, uncontrolled spasticity can induce abnormal postures, movements stiffness, and even muscle growth inhibition (14, 15).

Parents can help reduce spasticity through the application of thermal modalities, such as cold and heat. Cold therapy can be given with a cold pack; however, it should not be directly placed on the skin. The cold pack should be put in the towel. The cold pack should be applied for 5 min; then, it should be removed, and the skin should be dried. If a child’s skin is sensitive to cold, cold therapy should never be used (16). A heating pad increases muscle elasticity and causes the muscle to relax. Therefore, the heating pad should be used combined with stretching and exercises (17).

**Maintaining Joint ROM**

Children with neurologic diseases often receive passive stretching that is intended to maintain or increase their joint passive ROM (18). Passive ROM is performed in such a way that child’s parents move a part of the body, and the child has no role in the movement. Parents do not need to use much energy and expertise to do exercises. Therefore, all parents can use passive exercises for their children. As passive movements damage a child’s joints and muscles, passive ROM exercises should be executed until the child feels pain (19). Passive ROM exercises are suitable for children who have severe to moderate spasticity and joint stiffness and are still unable to control their movements as much as they should (20). Table 1 lists the most important passive movements.

**Table 1:** Passive Movements in Upper and Lower Limbs

**Upper limb movements**	**Lower limb movements**
Shoulder rotation	Hip rotations
Shoulder flexion and extension	Hip abductions
Shoulder horizontal abduction and adduction	Knee flexion and extension
Shoulder abduction and adduction	Ankle rotations
Elbow flexion and extension	
Wrist flexion and extension	
Wrist rotation	

**Avoiding Muscle Shortening with Stretching**

In children with neurological disorders, stretching exercises were performed to manage spasticity in the form of passive and active stretching, positioning, and isotonic and isokinetic stretching (21). Table 2 shows the most important stretching exercises for children with muscle stiffness. For the improvement of ROM, passive or active stretches could be applied by parents or as initial conservative treatments. Walking or any mobility after stretching interventions is important (22). 

**Table 2:** Stretching Exercises

**Upper limb movements**	**Lower limb movements**
Shoulder stretch	Hamstring stretch
Wrist stretch	Heel cord wall stretch
Elbow extension stretch	Seated heel cord stretch
	Quadriceps stretch
	Knee to chest stretch

**Maintaining Gross and Fine Motor Activities**

One of the most important steps in the rehabilitation procedure of children with neurologic disorders is the examination of the child’s physical development (23). According to the child’s ability, exercises should be daily performed by parents. Creping, rolling, quadruped, kneel standing, standing, walking, scribbling, coloring, drawing, writing, scissors skills, and any mobility that a child would do can be daily repeated (24). Parents can use weight cuffs at the end of the upper and lower extremities for the stimulation of proprioception sense for better control of movements (25). 

**Maintaining and Improving Balance Abilities**

Parents should implement balance exercises for all children at different mobility levels. Exercises, such as weight shifting, toe and heel walking, push-off and toe-off, using tilt board, standing on one leg, sitting and standing on an uneven surface, walking on the balance beam, and tandem walking, are helpful and should be daily performed 

## In Conclusion

to the aforementioned exercises, perceptual-motor training activities and sensory integration activities are also exercises that therapists can teach parents of these children through audio and video online communication.
